# Paraplegia as a Rare Clinical Presentation of Relapsed Acute Myeloid Leukemia

**DOI:** 10.7759/cureus.41421

**Published:** 2023-07-05

**Authors:** Haitham Osman, Israa Gism Elseed, Sheikheldein B Hussein, Naima Mando, Alaa A Alraheili

**Affiliations:** 1 Hematology, Prince Muhammad Bin Abdulaziz Hospital, Ministry of National Guard Health Affairs, Madinah, SAU; 2 Medicine, Prince Muhammad Bin Abdulaziz Hospital, Ministry of National Guard Health Affairs, Madinah, SAU; 3 Intensive Care Unit, Prince Muhammad Bin Abdulaziz Hospital, Madinah, SAU; 4 Internal Medicine, Prince Mohammed Bin Abdulaziz Hospital, National Guard Health Affairs, Madinah, SAU

**Keywords:** granulocytic sarcoma, paraplegia, chloroma, myeloid sarcoma, acute myeloid leukemia

## Abstract

Myeloid sarcoma, also known as granulocytic sarcoma or chloroma, is an extra-medullary accumulation of malignant myeloid blast cells, leading to a solid tumor formation. Herein, we report a rare presentation of a case with acute myeloid leukemia (AML), whose disease relapse was clinically evident as acute flaccid paraplegia with a certain sensory level. On thoracic spine magnetic resonance imaging (MRI), an epidural mass compressing the spinal cord at the level of the thoracic spine segment 4 (T4) was found. The mass histology confirmed the diagnosis of myeloid sarcoma.

## Introduction

Myeloid sarcoma is a collection of malignant myeloid blasts that form a solid mass that substantially changes the tissue architecture [1]. “Chloroma” is derived from a Greek word (chloros, meaning green), and is an alternative term for the tumor due to their frequent green appearance from myeloperoxidase abundance. It occurs in a few cases of acute myeloid leukemia (AML) and the accelerated phase and blast crisis phase of chronic myeloid leukemia (CML) [2]. It may also represent a disease progression in myelodysplastic syndrome (MDS) or myeloproliferative neoplasm (MPN), as both can progress to AML.

The site of chloroma cannot be predicted, and it can be in different sites, such as the soft tissue, lymph nodes, skin (leukemia cutis LC) [3], and the eyes (orbital chloroma), a tumor location mostly seen in children [3,4]. Chloroma can manifest as the first presentation in healthy individuals, who then are going to develop AML months to years later [4]; otherwise, it may occur at relapse in a known case of AML, as in this case report.

The presence of myeloid sarcoma (chloroma) is enough to treat a patient with the AML protocol even if the bone marrow is clear [5]. Chloromas have been reported both in de novo and AML recurrence cases [6]. It remains a unique entity in the revised World Health Organization (WHO) 2016 classification of myeloid neoplasm [7].

## Case presentation

A 20-year-old male was previously diagnosed with AML after presenting with fever, gum bleeding, cervical lymphadenopathy, and leukocytosis with a WBC count of 64,000 cells/uL. However, at that time, he did not suffer from any neurological, respiratory, or gastrointestinal symptoms. The diagnostic workup, including bone marrow examination and molecular cytogenetic, confirmed the diagnosis of AML with complex cytogenetic abnormalities: an addition of chromosomes 4 and 6 and loss of chromosome 13 (trisomy 4, trisomy 6, and monosomy 13), making him at a higher risk of AML.

The patient started chemotherapy with the 3+7 protocol, went into complete remission (CR1), and then received two cycles of consolidation chemotherapy with high-dose cytarabine arabinoside (HiDAC). Later, he was seen in a transplant center to prepare him for an allogeneic stem-cell transplant (Allo-SCT) after finishing full consolidation cycles but unfortunately, he was lost to regular follow-up. The family was contacted several times, but they did not come for the scheduled appointments.

Two months later, he presented in the emergency room with sudden-onset weakness and numbness involving both lower limbs, preventing him from walking or even standing. The patient stated that these symptoms started a few hours before his arrival at the emergency room but were preceded by a two-week history of upper back pain radiating to the anterior chest wall without a history of back trauma. There was no fever, cough, hemoptysis, shortness of breath, headache, neck pain, neck stiffness, or photophobia. Furthermore, he reported that he did not pass urine for the last 12 hours.

On examination, he was fully conscious and oriented to time, place, and person. There were palpable, enlarged, hard, non-tender left anterior and right postauricular cervical lymph nodes around 2 × 2 cm in diameter. There were no other affected lymph nodes. An oral exam showed mild gum hypertrophy. The cranial nerves exam revealed no facial asymmetry, and all cranial nerves were intact. Upper limbs neurological examination was normal, whereas the lower limbs examination revealed a flaccid weakness with a power of 0/5, areflexia, and loss of all sensations up to the nipples bilaterally. No tenderness over the spine could be appreciated on examination. The abdominal examination revealed no palpable liver and spleen, but there was a soft and tender palpable mass in the hypogastric area extending up to the umbilical region, which seemed to be a distended urinary bladder. Foley’s catheter was inserted, immediately draining around 1500 ml of urine. Urgent magnetic resonance imaging (MRI) of the dorsal spine was obtained, showing upper dorsal cord compression extending from T2 to T6 due to posterior extra-medullary epidural/subdural neoplastic mass. The cord was diminished in width (compressed cord) without signs of vascular thrombosis or intramedullary or extra-medullary hemorrhage.

**Figure 1:**
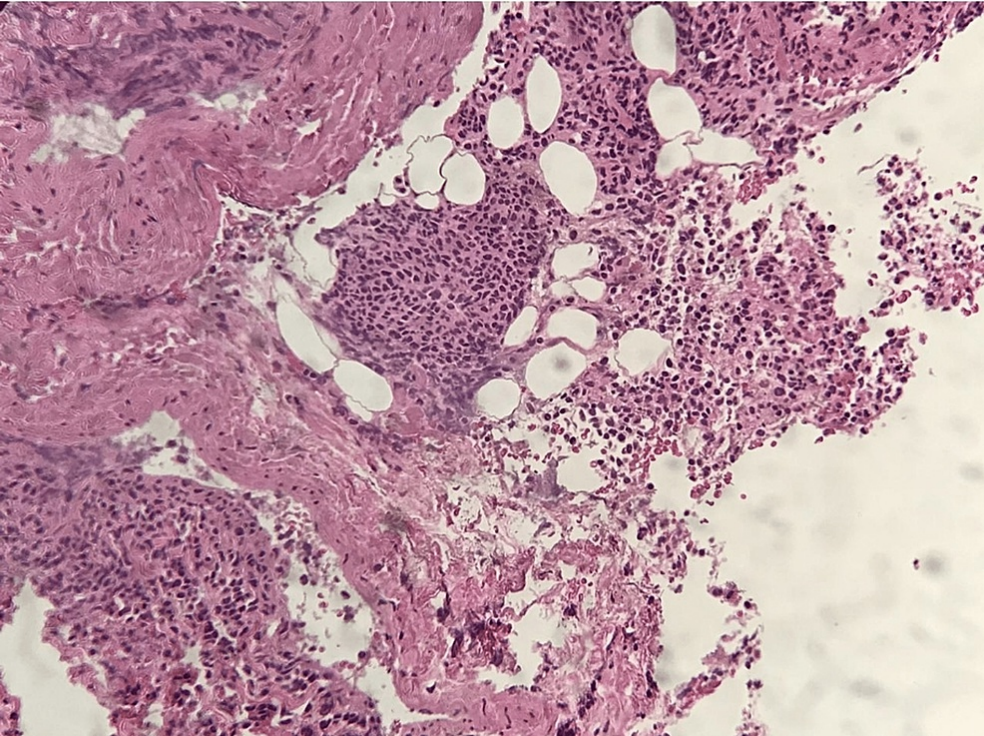
The higher magnification shows the epidural mass as a monotonous population of blast cells

**Figure 2:**
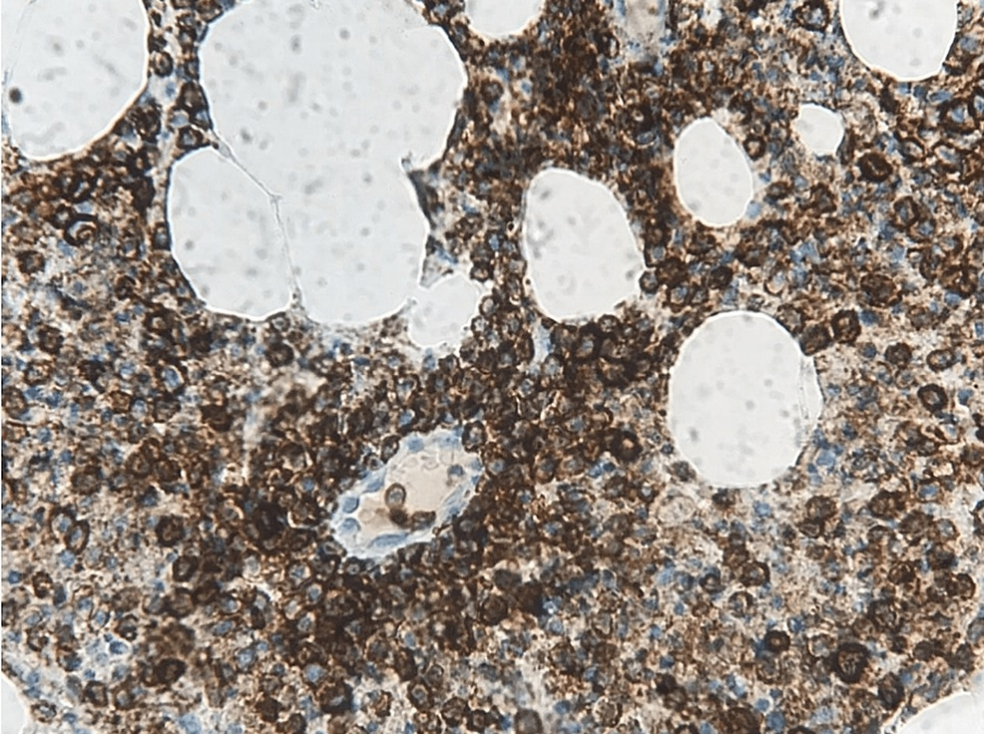
Stain positive for myeloperoxidase at low magnification

An urgent neurosurgical referral was made after starting dexamethasone 8 mg intravenously every eight hours. He was then taken to the operating room for spinal decompression surgery. The soft tissue mass in the epidural space compressing the spinal cord and extending from T2 to T6 was excised and sent for culture and tuberculosis polymerase chain reaction (TB PCR), which later came negative. The histopathology exam showed a mass comprising tumor cells consisting of neoplastic immature myeloid cells and necrosis (Figure 1). The neoplastic cells were described as large cells with abundant cytoplasm and large nuclei. They stained positively for myeloperoxidase immune stain and clusters of differentiation (CD) 43 and negatively for TdT and CD117 (c.Kit) (Figure 2). These results supported the diagnosis of myeloid sarcoma.

## Discussion

The abrupt onset that occurred within four to six hours of the neurological deficit indicated that the most likely cause of paraplegia was spinal artery occlusion with thrombus or para-spinal hemorrhage; however, MRI of the spine excluded both these causes. Other common causes of paraplegia with a sensory level, such as spinal tuberculosis (TB; Pott’s disease) or para-spinal abscess, were considered initially given that the patient was immune-compromised and on chemotherapy and was ruled out. The MRI findings are not specific in myeloid sarcoma [8] while histopathology is decisive.

Chloroma of the spine is usually extra-medullary, mostly located in the epidural space, as in this patient’s case, whereas intramedullary chloroma is extremely rare [8]. Extra-medullary involvement with myeloid sarcoma was associated with some clinical, morphological, and cytogenetic features that may help in predicting which patient is at risk of developing it. Monocytic and myelomonocytic M4 or M5 AML, as per the French-American-British (FAB) classification [9], which is the morphological AML classification, is commonly associated with myeloid sarcoma and more likely to form chloromas than other AML subtypes. Other sporadic CD markers, such as CD56, CD2, CD4, and CD7, are associated with myeloid sarcoma.

Certain cytogenetic abnormalities in the malignant myeloid cells are associated with myeloid sarcoma, including inv(16) and t(8;21), and some molecular mutations such as FLT3 mutations and MLL rearrangement. Trisomy 8 has been the most common cytogenetic association that may point to extra-medullary involvement in AML [10].

Clinically, the presence of lymphadenopathy, which is uncommon in myeloid malignancies but common in lymphoid malignancy, can be considered myeloid sarcoma of the lymph node with the cervical group of nodes being the most common site of extra-medullary involvement in AML [11]. Gingival involvement is another clinical finding pointing to extra-medullary involvement in AML. Both the latest clinical findings were evident in our patient's case.

A previous case report described an AML accompanied by thoracic epidural myeloid sarcoma in a 59-year-old man who initially presented with sudden-onset paraplegia, which is similar to our case. However, he had a white blood cell count at presentation of 2,300/μL, which is lower than our patient; thus indicating that high leukocyte count at AML presentation is not necessarily associated with developing chloroma [12]. On the other hand, gingival tissue is more likely to develop leukemic cell invasion because of endothelial adhesion molecules expression, which may increase leukocyte infiltration [13].

Furthermore, the association of AML with intraoral chloroma is extremely rare [14] and might be falsely diagnosed as an inflammatory lesion, including pyogenic granuloma or even peripheral giant cell granuloma [13].

## Conclusions

Unlike lymphoid leukemia, myeloid leukemic blast cells can form a solid mass that invades different organs and causes sudden disability such as in our case report. Early diagnosis and intervention may decrease morbidity and mortality. Myeloid sarcoma should be predicted in all AML cases, especially if special predictors are present, such as those we emphasized in the discussion of our case report, including morphological, cytogenetic, and immune-histochemical predictors.
